# Remote Assisted Home Dressing vs. Outpatient Medication of Central Venous Catheter (Peripherally Inserted Central Venous Catheter): Clinical Trial A.R.C.O. (Remote Assistance Oncology Caregiver)

**DOI:** 10.3390/nursrep14020110

**Published:** 2024-06-11

**Authors:** Paolo Basili, Ilaria Farina, Irene Terrenato, Jacopo Centini, Nina Volpe, Vanessa Rizzo, Laura Agoglia, Albina Paterniani, Pasquale Aprea, Prisco Calignano, Fabrizio Petrone, Gennaro Ciliberto

**Affiliations:** 1 Technical, Rehabilitation, Assistance and Research Direction, Vascular Access Specialist IRCCS Regina Elena National Cancer Institute, 00144 Rome, Italy; paolo.basili@ifo.it (P.B.); vanessa.rizzo@ifo.it (V.R.); 2 Department of Radiation Oncology, IRCCS Regina Elena National Cancer Institute, 00144 Rome, Italy; 3 Clinical Trial Center-Biostatistics & Bioinformatics, IRCCS Regina Elena National Cancer Institute, 00144 Rome, Italy; 4 Nursing, Technical, Rehabilitation, Assistance and Research Direction IFO, IRCCS Regina Elena, National Cancer Institute, 00144 Rome, Italy; jacopo.centini@ifo.it (J.C.); nina.volpe@ifo.it (N.V.); fabrizio.petrone@ifo.it (F.P.); 5 Nuova Sair, 00131 Rome, Italy; laura.agoglia10@gmail.com; 6 School of Nursing, IRCCS Regina Elena National Cancer, 00144 Rome, Italy; albina.paterniani@ifo.it; 7 Vascular Access Unit, IRCCS-Fondazione G. Pascale National Cancer Institute, 80131 Napoli, Italy; aprepas@gmail.com; 8 Department of Hematology, IRCCS-Fondazione G. Pascale National Cancer Institute, 80131 Napoli, Italy; priscos74@libero.it; 9 Scientific Directorate, IRCCS Regina Elena National Cancer Institute, 00144 Rome, Italy; gennaro.ciliberto@ifo.it

**Keywords:** PICC, cancer patient, telenursing, nursing education, quality of life

## Abstract

Background: Management of PICC dressing can be performed at home by the patient through adequate training and telenursing. This trial verifies that the incidence of catheter-related complications in home patients, assisted by telenursing, is not greater than that observed in outpatients. Methods: This clinical trial is composed of 72 patients with malignant tumors who underwent long-term chemotherapy with PICC insertion. They were randomly divided into an experimental group (33 cases) and a calibration group (39 cases). The control group received outpatient dressing for the PICC at the hospital, while the experimental group received a telenursing intervention about the management of the PICC. The incidence of catheter-related infections, the ability of self-management, and a rough cost/benefit estimation were compared between the two groups. This trial was performed according to the CONSORT 2010 checklist. Results: The two groups do not significantly differ in relation to age, sex, and PICCs in terms of the body side insertion, the type of dressing, and the agents used for cleaning. The analysis of the results showed that in the home-managed group, the clinical events reported during the connection were higher when compared with the outpatient group (*p* < 0.001). The patients in the homecare group developed frequent complications resulting from skin redness (*p* < 0.001). Conclusion: The use of telenursing for patient education in cancer centers can reduce nurses’ working time, improving the self-management capacity of patients with a long-term PICC. This trial was retrospectively registered with the Clinical Trial Gov on the 18 May 2023 with registration number NCT05880420.

## 1. Introduction

In clinical practice, “e-Health” was created as a new frontier of assistance. The term “e-Health” refers to the use of technological means to provide health-related information, resources, and services to improve patient care [1,2]. “e-Health” has promoted the expansion of homecare for alternative care pathways (e.g., telenursing, telemedicine), especially for cancer patients [3]. With the pandemic, the process of expanding alternative welfare services has seen rapid development to cope with the decline in new diagnoses because of the interruption of cancer screenings and the slowdown in diagnostic activities. For many patients, these slowdowns/interruptions in activity have caused them to switch from hospital to home nursing care. In Italy, mission 6 of the National Recovery and Resilience Plan states that 10% of patients over 65 are under a homecare regime. Some complementary objectives are foreseen, including the creation of a new organizational model and the implementation of telehealth in all its forms [4]. Cancer patients need central venous access according to the different types of chemotherapy and support drugs during the treatment [5]. The presence of a central vascular access could produce mechanical issues, such as thrombotic and infectious complications, that can undermine the patient’s health and the life of the catheter; therefore, the management of venous accesses is clinically relevant. Many studies have shown that adherence to rigorous protocols regarding catheter insertion and care significantly reduces complications [5,6,7]. The prevention of infections is strongly based on correct hand washing and on compliance with aseptic techniques (i.e., no-touch procedure) during exit-site dressing (i.e., cutaneous access point of the catheter). Patients with a PICC (peripherally inserted central venous catheter) are required to have weekly outpatient visits to ensure correct management of the medication [8]. Considering the lack of specialized centers in CVC (central venous catheter) management, mainly in peripheral Italian areas, nurses are committed every day to matching access to medicines with the other therapeutic needs of patients [9]. To obtain an improvement in the quality of life of the patient and their family members or caregivers during the SARS-CoV-2 period, a study was conducted to carry out medication with a PICC at home in a remote assistance regime with the possibility of training a voluntary caregiver chosen by the patient. In this way, it was possible to reduce the number of outpatient visits and show that the home group does not have a greater incidence of PICC-related complications. Each medication performed at home by the caregiver for the patient was followed up remotely by a nurse (tutor/video) trained for this process by the IGAV-TEAM (Implantation Outpatients and Vascular Access Management) staff. The aim of the study is to verify that the incidence of catheter-related complications in home patients, assisted by telenursing, is not greater than that observed in outpatients. In this work, we demonstrated that a family caregiver can safely perform procedures (e.g., washing and dressing PICCs) traditionally carried out by nursing staff in a hospital thanks to teleassistance and dedicated training. Furthermore, a technologically ad hoc infrastructure with the use of hand-free devices and point-of-care testing will allow for the domiciliation of outpatient activities, obtaining a positive return in terms of the cost–benefit balance.

## 2. Methods

### 2.1. Study Design

This clinical trial is composed of two arms: a single experimental group (homecare patients) and a calibration group (outpatients). The trial aims to test the hypothesis that the incidence of catheter-related complications in homecare patients is not greater than that observed in outpatients. The study complied with the guidelines outlined under the Consolidated Standards of Reporting Trials (CONSORT) checklist.

### 2.2. Patients

This study involved the enrollment of 72 adult cancer patients and their caregivers from two hospital clinical sites across Italy. The patients obtained a new PICC implantation or had their PICC implanted for no longer than 15 days. Before the trial started, ethical approval was given by both of the clinical locations. patients were asked to take part in the trial if they satisfied the following inclusion criteria: Patients were excluded from the study if they were unable to meet the above criteria and if they did not agree to give access to their case notes. All patients gave written informed consent following written and verbal information about the project.

### 2.3. Caregivers

Adult caregivers were eligible if they met the following criteria: (a) shared the patient’s choice; (b) were available during online appointments with a tutor/video in the way described in the methods and tools; and (c) participated in a small 3 h training course at the reference center.

### 2.4. Health Professional

Fourteen trained nurses were employed for the teleassistance course. The personnel were selected during the training course, and one of the parameters was the willingness of the professionals to participate in this project. The procedures and records were continuously respected through verbal communication and the recording of deliveries. The audit was recorded in the research quality control form, but the perceptions of these health professionals were reported elsewhere.

### 2.5. Intervention Group Study Procedure

The telenursing intervention program used in this study organized a training program for caregivers by creating an integrated remote assistance system to observe and assist family caregivers who have received specialized nursing training about the home management of medium-/long-term venous access (CVC/PICC). In the telenursing group, the trained caregiver (active surveillance) assisted by the expert nurse tutor/video recorded the adverse event. In the outpatient group, active surveillance was conducted by the nurse. The Supplementary Materials provide an extensive description of the intervention group procedure.

### 2.6. Primary Outcome

The primary outcome was to compare the incidence of complications in a PICC’s management both in the outpatients (calibration group) and the homecare patients (experimental group). The hypothesis was as follows: the incidence of catheter-related complications in the home management group will not be superior to that observed in the outpatient group.

### 2.7. Secondary Outcome

The secondary outcome was related to the costs/benefits of telemedicine. No specific analysis was carried out, but a rough estimation of costs was made.

### 2.8. Sample Size Calculation

The same amount of data were collected from the experimental group and from the calibration group. The sample size (related to medications) was estimated to be approximately 200 individuals, but due to issues related to a shortage in PICC device supply (a four-month supply delay) and a shortage in point-of-care (POC) connection device supply (a two-month supply delay), the sample enrolled was 72 patients. Moreover, since the study was conducted during the pandemic, there was a shortage of nursing staff for some periods and the impossibility of managing some patients affected by COVID-19.

### 2.9. Randomization

Using a computer-generated randomization sequence, patients were randomized in a ratio of 1:1 to each arm, but since six patients in the experimental group had connection issues as a result of connection kit delivery delays, the two arms differed in size.

### 2.10. Statistical Analysis

Descriptive statistics were computed for all variables of interest. Pearson’s Chi-square test, or Fisher’s exact test when appropriate, was used to compare the two groups of intervention. A *p*-value of <0.05 was considered statistically significant. Data double-checking and cleaning were conducted from October 2022 to December 2022. Data were analyzed with SPSS version 21.0 for Windows (SPSS Inc., Chicago, IL, USA).

## 3. Results

The data were collected between July 2021 and September 2022. The trial was delayed by 40 weeks from its approval by all the stakeholders. We designed a dataset for the data collection, divided into the two groups analyzed in the protocol. The records inserted were demographic data, disease-related data, PICC insertion-related data, and PICC complications data, such as phlebitis, CRBSI (Catheter-Related Bloodstream Infection), all BSIs (bloodstream infections), infiltration, occlusion, dislodgement, and venous (local) infection. The data included missed appointments, absence or difficulty due to a bad internet connection, and caregiver difficulties. During the trial, 313 connections/interventions were recorded for the 33 patients enrolled in the home group; in the outpatient group, 39 patients were enrolled, and 374 therapies/interventions were completed. During the analysis of the data from the homecare group, 89 events were recorded, 27 of which (8.6%) were related to a caregiver experiencing difficulty activating the electronic device and 24 (7.7%) to connection problems. Among the 89 events, 63 (20%) were related to difficulty in taking a photograph and 26 (8.3%) to POC device malfunction. The clinical characteristics of the patients are reported in Table 1.

There were no clinically significant differences between the home group and the outpatient group in relation to age, sex, body side insertion, type of dressing, or agents used to clean the PICC at the end of the infusion. A total of 687 interventions in 72 patients were performed during the study period. Table 1 shows no significant differences between the two groups in terms of vein quality, anatomic location of the insertion site, treatment prescribed, types of dressing used, or materials used for auxiliary fixation. If the PICCs were washed, the bolus drug was prepared in a 0.9% saline solution. This helps to block the PICC after the infusion is completed.

The analysis of the clinical results showed that the total number of clinical event (i.e., skin redness, partial occlusion, etc.) registrations was 40 (5.8%) during the study: 27 (8.6%) were from the homecare group and 13 (3.5%) from the outpatient group.

We reported all the adverse events in each intervention group according to item 19 of the CONSORT guidelines reported by Ioannis et al. [10]. The adverse events in the two groups included exit site redness, skin redness, deep vein thrombosis, total displacement, and partial occlusion. The percentage is similar between the two groups in the case of exit site redness (1.6% outpatient vs. 2.2% telenursing) and total displacement (0.8% vs. 4.8%) and quite different in the case of skin redness (0.8% vs. 4.8%). We noticed no deep vein thrombosis and no partial occlusion in the telenursing and outpatient groups, respectively.

The risk of exit site flushing between the two groups was not significant (*p* = 0.545), with an absolute risk difference of % (ARD). The data analysis found no significant difference in deep vein thrombosis (PV) (*p* = 1.000). None of the patients in the two groups reported phlebitis, partial dislocations, or blood infections. The incidences of exit site redness, suspected DVT (deep vein thrombosis), total displacement, and partial and total occlusion were similar between the two groups (see Table 2).

The travel time from home to the hospital and from the hospital to home is one of the most influential factors if the patients are assisted at home. The journey times are not directly proportional to the distance traveled due to logistical problems related to car traffic or public transport.

A reasonable, even if approximate, cost estimate leads us to consider an average saving for patients between EUR 100 and 200. This considers 10 medications (10 weeks) envisaged for enrollment in the project and includes only the costs of transport (petrol and any road tolls).

## 4. Discussion

The purpose of this study was to show that cancer patients with a PICC monitored by telenursing (i.e., the caregiver at home is under the guidance of skilled nurses) do not experience a greater incidence of catheter-related infections than patients who obtain care in an outpatient clinic. Skin redness was one of the possible PICC care consequences that patients in the home group reported more frequently than those in the outpatient group. However, this complication was quickly treated with the tutor’s/video nurse’s assistance. The findings of this investigation are in line with a review conducted by Duwadi et al. [11], which found that the majority of catheter-related issues can be avoided with adequate training in PICC care and management. In the outpatient group, trained caregivers carried out the reporting of events; however, in the home patient group, a higher percentage of occurrences were reported. This difference in reporting rates might be related to the inexperience of the caregivers in the second group: the caregiver, due to inexperience and insecurity, tends to overreport adverse events. This result is consistent with findings in the literature about family caregivers’ self-efficacy (or confidence) in providing homecare [12]. Telenursing is one way to address the issue of continuity of care, which can minimize clinic visits and lower the risk of infection for cancer patients. Our telemonitoring service makes it possible to organize adequate remote assistance between nursing staff and caregivers/family, answering every question about the clinical condition, observing the progress of complications related to the PICC, and suggesting how to prevent them. Furthermore, contact with experienced nursing staff helps avoid feelings of abandonment and stress in restricted conditions such as the pandemic period [13,14]. Other non-economic but even more relevant considerations concern the comfort of the cancer patient. Traveling even a few kilometers can take a long time due to traffic, and the patient’s conditions are not always optimal to deal with it serenely. In a study by Yan et al. [15], it was analyzed how nursing care via mobile internet for patients suffering from respiratory pathologies is important as it reduces the exposure of these fragile patients to the risk of contracting COVID-19 in crowded places such as hospitals. Since cancer patients with PICCs are highly susceptible to COVID-19, the waiting times inside the hospitals (which are not assessable with certainty but can go beyond even two hours) for outpatient care are detrimental. The risk of contagion, especially in the pandemic period, could be reduced by the telenursing service and through the use of “e-Health”. By using videoconferencing to provide services, telenursing decreases or eliminates the need for patients to travel, and it boosts nurses’ productivity since they may see more patients in a shorter amount of time. In the United States, a scheme designed by Dimmick et al. [16] to replace home visits with videoconferencing telemedicine saved 40,000 min of travel time in just 14 months and more than doubled the capacity of nurses. Song et al. [17] described how health education can help to promote healthy behavior among patients suffering from a PICC malignancy and reduce their complications in device management. The reason for this is the information provided to PICC wearers on the measures and behaviors to be adopted in case of complications. Remote assistance provides health education on improving one’s ability to take care of oneself. It makes the patient an active participant in treatments useful for their own health through the learning provided to the caregiver [18,19,20]. This connection creates a remote help relationship between the nurse, patient, and caregiver, which favors continuity of care in the area [21,22]. During the pandemic period, it was possible to accelerate the development of reliable and accessible local medicine [23]. Schaafsma et al. [24] found that the health service would save CAD 65,520 (USD 49,584.14) per year in travel expenses if telemedicine sessions replaced in-person sessions [25,26,27]. In our study, we confirmed, by a rough estimation, that money would be saved in the case that telemedicine was adopted, even if we did not report all the useful metrics (e.g., working time, staff cost, etc.) for calculating healthcare spending.

## 5. Relevance to Clinical Practice

Telenursing is an alternative to the classic assistance system linked to a patient’s hospitalization. This system allows a reduction in the time required for diagnosis and the identification of the correct therapy. Moreover, it improves the levels of assistance by reducing the number of examinations and amount of traveling, with considerable savings in time and money, especially in cancer patients who have a weakening of the immune system that is associated with comorbidities, with a marked improvement in their quality of life. Telenursing is highly professionalizing for nursing practice as it allows for the creation of relationships with the user in phases of health education and even in moments of care. The nurse’s profile should have new skills and new responsibilities with new professional independence.

## 6. Limitations

This clinical trial was conducted in two hospitals located in different geographical locations in Italy. The hospitals have a specialist reputation for characterizing the diagnosis and treatment of tumor pathologies. This trial was registered in the Clinical Trial Gov retrospectively to patient enrollment. At the start of the study, the sponsors of this study were unfortunately not aware of the policy of the International Committee of Medical Journal Editors (ICMJE), which requires the prospective registration of all interventional clinical trials. As soon as we learned of this policy, we registered the trial, and we tried to avoid any bias in the selective outcome. Moreover, the trial was conducted according to the ICH GCP and after ethics committee approval. The results should be interpreted while considering some study limitations. First, because of the small sample size due to issues related to the shortage in PICC device supply and POC connection device supply, the number of patients calculated in the power analysis was not reached. However, the training on the observation of the PICC insertion site and the quality control of the procedures, also implemented by the technological system made available to patients and caregivers, reduced the possibility of errors related to patient management and assistance.

## 7. Conclusions

Telenursing is a form of remote assistance that guarantees the periodic monitoring of a patient’s general conditions. Thanks to telenursing, it is possible to fill the care gap that is created when a patient leaves the hospital (i.e., hospital discharge). The guidelines for PICC management and infection prevention contain recommendations that can be taught to patients and caregivers. One of the limitations of the clinical study is related to the retrospective registration from the date of patient enrollment. Furthermore, the sample size is smaller than estimated due to the problems encountered; therefore, the understanding of caregiver compliance in the medium term was not studied. In future research, we may expand the sample size, which could lead to more generalizable conclusions. Moreover, an in-depth cost analysis will be carried out to analyze and evaluate the impact of the two interventions.

## Figures and Tables

**Table 1:** Patient and PICC characteristics.

	**Outpatient Intervention** **(*n* = 39)** ***n* (%)**	**Telenursing Intervention** **(*n* = 33)** ***n* (%)**
**Age (years)**		
Mean (min–max)	55.5 (27–75)	56.7 (30–82)
**Gender**		
Female	26 (66%)	23 (70%)
Male	13 (34%)	10 (30%)
**Type of cancer**		
Breast	19 (48.7)	6 (18.0)
Colorectal	9 (23.1)	10 (30.0)
Lung	2 (5.1)	4 (12.1)
Myeloma	2 (5.1)	1 (3.0)
Linfoma	2 (5.1)	4 (12.1)
Head and neck	2 (5.1)	0
Sarcoma	1 (2.6)	2 (6.0)
Gastric/esophagus	1 (2.6)	2 (6.0)
Pancreatic	1 (2.6)	0
Ovarian	0	2 (6.0)
Endometrial	0	1 (18.0)
**Bodyside insertion**		
Left	12 (30.0)	6 (18.0)
Right	27 (70.0)	27 (82.0)
**Types of dressing**		
**Polyurethane**	39 (100)	33 (100)
**Agents used to clean the PICC at the end of the infusion**		
Pre-filled syringes with saline solution	39 (100)	33 (100)
	**Outpatient Intervention** **(*n* = 39)** ***n* (%)**	**Telenursing Intervention** **(*n* = 33)** ***n* (%)**
**Age (years)**		
Mean (min–max)	55.5 (27–75)	56.7 (30–82)
**Gender**		
Female	26 (66%)	23 (70%)
Male	13 (34%)	10 (30%)
**Type of cancer**		
Breast	19 (48.7)	6 (18.0)
Colorectal	9 (23.1)	10 (30.0)
Lung	2 (5.1)	4 (12.1)
Myeloma	2 (5.1)	1 (3.0)
Linfoma	2 (5.1)	4 (12.1)
Head and neck	2 (5.1)	0
Sarcoma	1 (2.6)	2 (6.0)
Gastric/esophagus	1 (2.6)	2 (6.0)
Pancreatic	1 (2.6)	0
Ovarian	0	2 (6.0)
Endometrial	0	1 (18.0)
**Bodyside insertion**		
Left	12 (30.0)	6 (18.0)
Right	27 (70.0)	27 (82.0)
**Types of dressing**		
**Polyurethane**	39 (100)	33 (100)
**Agents used to clean the PICC at the end of the infusion**		
Pre-filled syringes with saline solution	39 (100)	33 (100)

**Table 2:** Study outcomes by treatment group, *n* (%).

	Outpatient Interventions	Telenursing Intervention	*p*-Value *
	(*n* = 374) *n* (%)	(*n* = 313) *n* (%)	
**Primary Outcome for Protocol Analysis**			
**Clinical Event Reporting**	13 (3.5%)	27 (8.6%)	<0.001 °
**Exit site redness**	6 (1.6%)	7 (2.2%)	NS **
**Skin redness**	3 (0.8%)	15 (4.8%)	<0.001 ^
**Deep vein thrombosis**	1 (0.3%)	0 (0%)	NS
**Total displacement**	3 (0.8%)	1 (0.3%)	NS
**Partial occlusion**	0 (0.0%)	3 (1.0%)	NS
**Total occlusion**	0 (0.0%)	1 (0.3%)	NS

We reported only statistically significant *p*-values: * significant; ** not significant; ° Chi-squared test; ^ Fisher’s exact test.

## Data Availability

Data will be made available by contacting the first author and corresponding author upon reasonable request.

## Supplementary Materials

The following supporting information can be downloaded at: https://www.mdpi.com/article/10.3390/nursrep14020110/s1, File S1: Intervention group study procedure.

## Public Involvement Statement

There has been no public involvement in any aspect of this research.

## Guidelines and Standards Statement

This manuscript was written according to the CONSORT 2010 checklist for RCT.
