# Supplementary material for: Remote Assisted Home Dressing vs. Outpatient Medication of Central Venous Catheter (Peripherally Inserted Central Venous Catheter): Clinical Trial A.R.C.O. (Remote Assistance Oncology Caregiver)
Source: Nurs Rep. 2024 Jun 11;14(2):1468–76. doi: 10.3390/nursrep14020110 (PMC11206720; doi:10.3390/nursrep14020110)
Supplement: Supplementary file 1 [file nursrep-14-00110-s001.zip › nursrep-2765238-supplementary.pdf]

Supplementary Materials:

Intervention group study procedure

The project involved the development and testing of two technological platforms, used for the home management of medium and long-term venous access in cancer patients. The two platforms are dedicated to patient care at their place of hospitalization and caregiver support via wearable device, respectively.

The technological platform consists of the following elements:

• A central HUB with functions of:

1. Authentication server

An authentication server provides a network service to authenticate the credentials, typically account names and passwords. When a customer submits a valid set of credentials, receives a cryptographic ticket that can later use to access various services.

2. Signaling servers

A server that allows you to coordinate communication between connecting devices and send them control messages.

3. Turn server

“Traversal Using Relays around NAT” (TURN) is a protocol that supports traversal of Network Address Translator (NAT) or firewalls for multimedia applications. This server allows communication between devices that connect via NAT from complex network structures.

4. Media servers

A server dedicated to store various digital media (in our case: video, audio and images). At the hospitals, one or more workstationsare present. Point-of-care terminals are used at patients' homes, consisting of multiple types of devices (industry-standard smartglasses) of two or more models with different characteristics. The point-of-care (POC) devices used are smartglasees designed to have hands-free operation, i.e. the ability to use the device "hands-free" is among the most important properties for the application because the caregiver is responsible that all operations are carried out in high hygiene conditions: the material is disposable, sterilized and must be treated with the utmost care. The platform has provided a shared and synchronized calendar. The access is granted to users and healthcare personnel, The platform provided a shared and synchronized calendar. Access is allowed to users and healthcare personnel, the organization of visits will be agreed.

Remote assistance services can be divided into 3 bands, distinguished by a code:

Red: inexperienced users or with objective operational difficulties related to the patient;

Yellow: averagely expert users who are not expected to encounter operational difficulties but who may require "motivational" support;

Green: expert and essentially self-sufficient users for whom the operator will simply have to carry out the routine procedures required by the protocol.

The healthcare personnel has the possibility to manage multiple interventions at the same time, after evaluating the level of autonomy of each caregiver or the particular situation of the patient (red, yellow or green codes). A "red code" will usually have the exclusive attention of the operator, while there may be greater or lesser parallelization in the other cases.

The user is recognized only after entering username and password. There is an additional one time password(OTP) which can be delivered (or generated) via: SMS on the mobile phone; email on the company mailbox; using an authentication app (i.e. Google Authenticator); facial recognition.

The caregiver training course, carried out by the IGAV (Implantation Outpatients and Vascular Access Management) experienced nurses, was divided into: 1.5 hours of theory, 1.5 hours of practical exercise on a manikin simulator, instruction on the use of the POC connection system (smartglasses or other hands-free devices) and the face-to-face video programme to book the tutor/video on how to carry out the medication. During caregiver training, staff reserve the right to withdraw patient enrolment in the home group if they do not feel the caregiver can handle the PICC infusion system in the most appropriate manner. At the end of the course, the nursing staff in charge of the IGAV will evaluate the actual ability demonstrated by the caregiver to handle the medication.

The patient can connect with the tutor/video through the internet connection guaranteed by a data SIM or a WIFI connection at home. The scheduled time slots were from Monday to Friday with the following times: morning from 9.30 to 11.00 (five appointments lasting 30 minutes each); afternoon from 16.00 to 19.00 (six appointments lasting 30 minutes each). The booking was completely free of entering sensitive data and took place in the following way: the patient accesses to the booking portal by using an App installed on the POC connection system that has been provided to him when he joined the trial to reserve the tutor. For the first 2 medications, the relationship between tutor and patient/caregiver is expected to be 1 to 1 and therefore, the patient will have to book on a free red slot; for the next two medications (the third and fourth) the tutor video ratio will be 1 to 2, therefore the tutor will follow 2 patients simultaneously on the screen with no possibility of video or audio mixing between the 2 patients. In this case, the medication was reserved on the yellow slot; for the remaining 6 medications the tutor video ratio is 1 to 4, therefore the tutor will follow 4 patients on the screen at the same time; in this case the medication was booked on the green colored. According to the tutor's feedback, the patient could be directed towards an extension of the red or yellow links or return to more individual links if the caregiver would demonstrate uncertainty (up to the exit of the study if no longer considered suitable); if minor complications were suspected (e.g. slight reddening of the exit site), the tutor can decide for more assistance. After at least one first outpatient medication (24/72 hours), the patients included in the experimental group, carry out the PICC's management at home with the support of the chosen caregiver, previously instructed and trained, guided by

an expert operator virtually connected through the monitor/video ("tele-nursing"). The medications were carried out at defined times through a specific reservation, according to the indications and instructions given (with relative telematic assistance), every 7 days up to a maximum of 10 weeks. Patients/caregivers have been provided with all the necessary materials, instructions and support. Patients included in the calibration group had one outpatient dressing every week for a total of 10 weeks as per standard procedure. The tutor/video must: request a close supplemental dressing at his/her discretion; ask to the patient to go to the reference center for complications that cannot be managed otherwise; communicate the procedure for suspecting major complications (thrombosis, infections or significant bleeding) by inviting the patient either to the reference center, if accessible, in a very short time according to the opening hours or to the emergency room and following the operating procedures in his possession attached to the study design. At the beginning of the dressing, the tutor/video will take a photo framing the dressing only in a "narrow" way and the same will happen at the end of the application of the new dressing. These images will be kept respecting all forms of privacy. The kit delivered in the experimental group included a backpack containing a POC connection system with a smartphone (platform EYE 4 CARE) kit and 5 kits/sterile materials for dressings. The next 5 kits have been sent to the patient's home. Upon delivery of the complete POC system or smartglasses (platform EYE 4 CARE), the patient or their caregiver must sign a delivery sheet allowing them to return the system to its original conditions.
